# Inhibition of store-operated calcium channels by N-arachidonoyl glycine (NAGly): no evidence for the involvement of lipid-sensing G protein coupled receptors

**DOI:** 10.1038/s41598-020-59565-4

**Published:** 2020-02-14

**Authors:** Aykut Deveci, Jessy Hasna, Alexandre Bouron

**Affiliations:** 1 Université Grenoble Alpes, CNRS, CEA, IRIG-LCBM, 38000 Grenoble, France; 2 Present Address: Genetics and Chemogenomics UMRS-1038 INSERM-CEA-UGA, CEA, 17 rue des Martyrs, 38054 Grenoble, France

**Keywords:** Lipid signalling, Cellular neuroscience, Ion channels in the nervous system

## Abstract

N-arachidonoyl glycine (NAGly) is an endogenous lipid deriving from the endocannabinoid anandamide (AEA). Identified as a ligand of several G-protein coupled receptors (GPCRs), it can however exert biological responses independently of GPCRs. NAGly was recently shown to depress store-operated Ca^2+^ entry (SOCE) but its mechanism of action remains elusive. The major aim of this study was to gain a better knowledge on the NAGly-dependent impairment of SOCE in neurons of the central nervous system (CNS) from mice. First, we examined the expression of genes encoding for putative lipid sensing GPCRs using transcriptomic data publicly available. This analysis showed that the most abundant GPCRs transcripts present in the cerebral cortices of embryonic brains were coding for lysophosphatidic acid (LPA) and sphingosine-1 phosphate (S1P) receptors. Next, the presence of functional receptors was assessed with live-cell calcium imaging experiments. In primary cortical cells S1P and LPA mobilize Ca^2+^ from internal stores via a mechanism sensitive to the S1P and LPA receptor antagonists Ex26, H2L5186303, or Ki16425. However, none of these compounds prevented or attenuated the NAGly-dependent impairment of SOCE. We found no evidence for the requirement of lipid sensing GPCRs in this inhibitory process, indicating that NAGly is an endogenous modulator interfering with the core machinery of SOCE. Moreover, these data also raise the intriguing possibility that the depression of SOCE could play a role in the central effects of NAGly.

## Introduction

N-arachidonoyl glycine (NAGly) is a lipid deriving from the endocannabinoid anandamide (AEA). It is naturally present in various tissues and organs like the brain^1,2^ but the physiological functions exerted by NAGly in the neural system are not yet fully characterized. This endogenous bioactive molecule influences pain perception and displays analgesic properties^1,3–5^. This led to the hypothesis that NAGly could be a natural modulator of pain^6^. The analgesic actions of NAGly have been studied principally on dorsal root ganglia (DRG) neurons and dorsal horn neurons from spinal cord slices^7^. For instance, NAGly inhibits low threshold voltage-gated Ca^2+^ channels (Cav3) in DRG neurons^8^ and the glycine uptake transporter GLYT2 in dorsal horn neurons, which contributes to enhance inhibitory glycinergic synaptic transmission in these cells^7^. NAGly has also been shown to depress excitatory NMDA-dependent synaptic transmission^7^. The effects of NAGly on neurons of the brain have however been much less characterized. In primary cortical neurons NAGly releases Ca^2+^ from the endoplasmic reticulum (ER), potentiates the passive leakage of Ca^2+^ out of the ER, and impairs the store-operated Ca^2+^ entry (SOCE)^9^.

One key issue when addressing the question of the cellular effects of NAGly is to determine whether it interferes directly with the activity of its targets or recruits dedicated G-protein coupled receptors (GPCRs) linked to downstream intracellular signaling cascades. NAGly has been proposed to act as an agonist of some orphan GPCRs like GPR18^10^, GPR55^11^, and GPR92^12^. It is however worthy of note that NAGly can influence the activity of some effectors without the requirement of GPCRs. This is for instance the case for the NAGly-dependent regulation of voltage-gated Ca^2+^ channels and Na^+^/Ca^2+^ exchanger activity^8,13,14^.

SOCE is an important physiological Ca^2+^ route of the plasma membrane. It is activated in response to the depletion of the ER Ca^2+^ stores^15^ and involves distinct actors: stromal interacting molecules (STIM1–2) and Orai1–3 channels^16–18^. STIM are ER resident proteins that seem to function as Ca^2+^ sensors^19^. The depletion of the ER Ca^2+^ stores governs the molecular interaction between STIM and plasma membrane Ca^2+^ channels of Orai type that are responsible for the SOCE response^17,18^. Recent studies identified NAGly as a potent inhibitor of SOCE in various cell lines (NIH-3T3 fibroblasts, human endothelial cell line EA.hy926, rat pancreatic β-cell line INS-1 832/13, rat basophilic leukemia cell line RBL-2H3) and in primary cultured neural cells^9,20,21^. Two hypotheses were put forward to explain the NAGly-dependent depression of SOCE^20^: (1) direct disruption of the coupling between STIM and Orai, or (2) recruitment of an intracellular signalling cascade activated downstream to NAGly-sensitive receptors and regulating negatively SOCE activity. The aim of the present work was to verify whether a lipid sensing GPCR is contributing to the NAGly-induced impairment of SOCE in cortical neurons. First, we analyzed a recent publicly available transcriptomic dataset obtained by RNAseq^22^ to characterize the expression of genes encoding for putative lipid sensing GPCRs in the cerebral cortices of embryonic mice. The most abundant transcripts were coding for lysophosphatidic acid (LPA) and sphingosine-1 phosphate (S1P) receptors. After having checked the presence of functional receptors, the contribution of LPA and S1P receptors to the NAGly-dependent inhibition of SOCE was evaluated using a pharmacological approach.

## Material and Methods

### Animal and ethical statement

C57Bl6/J (stock #000664) mice were obtained from the Jackson Laboratory (USA). They were housed in a temperature-controlled room under a 12 h light–12 h dark cycle with ad libitum access to food and water. An environmental enrichment consisting in the addition of tunnels was provided for all animals in accordance with the Animal Welfare Committee of the CEA Grenoble. Experimental procedures were approved by the animal care committee of the CEA’s Life Sciences Division (CETEA, A14-006). Experiments were conducted in compliance with the French legislation and the European Community Council Directive of 24 November 1986 (86/609/EEC).

### Primary cultures of cortical neurons

Cells were dissociated from cerebral cortices collected from embryonic (E13) mice (with the vaginal plug as E0) according to^9,23,24^. Briefly, tissues were placed in a 1.5 mL Eppendorf tube containing 1 mL of an ice-cold Ca^2+^- and Mg^2+^-free Hank’s solution supplemented with 33 mM glucose, 4.2 mM NaHCO_3_, 10 mM HEPES, and 1% penicillin/streptomycin. Cells were isolated by a mechanical trituration of the medium containing the cerebral cortices. The cell suspension was filtered through a 40-µm cell strainer before plating the cells on 16 mm ∅ glass coverslips. They were kept in a Neurobasal medium supplemented with B27 (2%) and glutamine (500 µM) and maintained in a 5% CO_2_ atmosphere at 37 °C. All the experiments were conducted on cells kept 2 or 3 days *in vitro*.

### Calcium imaging experiments with Fluo-4

The culture medium was removed and replaced by a saline containing (in mM) 150 NaCl, 5 KCl, 1 MgCl_2_, 2 CaCl_2_, 5.5 glucose, 10 HEPES (pH 7.4, NaOH). LPA- and S1P-induced Ca^2+^ responses were analyzed with Fluo-4. Cells were incubated with 5 μM Fluo-4/AM for 20 min following procedures described previously^23,24^. Images were obtained by a CCD CoolSnap HQ2 camera (Princeton Instruments, Roper Scientific, France) mounted on an inverted Zeiss A1 microscope (Carl Zeiss, France). Cells were excited at 470 nm and emission was collected at 525 nm using a DG-4 wavelength switcher (Princeton Instruments, Roper Scientific, France). MetaFluor (Universal Imaging, Roper Scientific, France) was used for image acquisition and analysis. All experimental procedures were conducted at room temperature. Time-lapse changes in Fluo-4 fluorescence intensity were collected at a frequency of 0.2 Hz from 30–45 cell bodies per dish and analyzed off-line by defining regions of measurements. Results were expressed as F/F0, with F being the fluorescence at each time point and F0 being the mean baseline fluorescence that was monitored at the beginning of each experiment for 1 min before the addition of any substance. Culture dishes were discarded at the end of the recording and never re-used. A positive LPA (or S1P)-induced calcium response was determined as one F/F0 greater than 0.02 that develops within 50 s upon the application of the agonist. Fluo-4 responses were measured as area under curve (AUC).

### Calcium imaging experiments with Fura-2

The fluorescent Ca^2+^ probe Fura-2 was used to study store-operated Ca^2+^ entry (SOCE). The experimental conditions and setup were as above except that cells were incubated with 2.5 µM Fura-2 for 20 min at room temperature. They were then washed twice and kept in a Fura-2-free saline solution for >12 min at room temperature. A dual excitation at 340 and 380 nm was used and emission was collected at 515 nm. Images were acquired at a frequency of 0.2 Hz and analyzed off-line. The classical “Ca^2+^ add-back” protocol was used to study SOCE. Cells were bathed in a nominally Ca^2+^-free saline containing (in mM) 150 NaCl, 5 KCl, 3 MgCl_2_, 5.5 glucose, 10 HEPES (pH 7.4, NaOH). SOCE activation was triggered by depletion of the ER Ca^2+^ pool with 200 nM thapsigargin, which induced a transient elevation in intracellular Ca^2+^ concentration before re-admission of 2 mM external Ca^2+^. SOCE responses were analyzed in cells generating a rapid Ca^2+^ rise upon the application of a depolarizing saline containing 90 mM KCl. In cultures of embryonic cortical cells, KCl responding cells are identified as neurons whereas KCl-unresponding cells are considered as non-neuronal cells^25^. The depolarizing (K^+^ rich) medium had the following composition (in mM): 65 NaCl, 90 KCl, 1 MgCl_2_, 2 CaCl_2_, 5.5 glucose, 10 HEPES (pH 7.4, NaOH). Ca^2+^ changes as a function of time were expressed as delta ratio F340/F380 whereas total Ca^2+^ responses were measured as area under curve (AUC).

Stock solutions of Ex26, Ki16425, and BTP2 were prepared in dimethyl sulfoxide (DMSO). Methanol and ethanol were used for preparing stock solutions of S1P and NAGly, respectively. These stock solutions were diluted at least 1000-fold into the recording saline immediately before use so that the final concentration of vehicle never exceeded 0.1%. Control experiments were performed with DMSO, ethanol and methanol alone. None of the solvent altered cytosolic Ca^2+^ signals, at least at the concentrations used.

### Analysis of gene expression by RNAseq

The RNASeq gene expression data derive from^22^. Raw fastq files are publicly available and can be found on the GEO repository under accession number: GSEXXX.

### Data and Statistical analysis

Each experimental condition as well as its appropriate control were tested on the same batch of primary neuronal cell cultures. For the Ca^2+^ imaging experiments, all experiments were done ≥3 times (e.g. with ≥3 distinct biological samples) using distinct dishes from different batches of cells (e.g. from distinct pregnant mice). Data are presented as means ± standard error of the mean (SEM) with *n* being the number of biological replicates. SigmaPlot (version 10.0, Systat Software) and SigmaStat (version 3.5, Systat Software) were used for plotting graphs and statistical analysis, respectively. Differences between several groups of cells were tested using one-way analysis of variance (ANOVA) followed by a Bonferroni’s *post hoc* test. A *P* value < 0.05 was considered statistically significant.

### Materials

Fluo-4/AM, Fura-2 and tissue culture media were from Molecular Probes (Invitrogen, France). N-arachidonoyl glycine (NAGly) was from Tocris (Bio-Techne, France). All the other reagents were obtained from Sigma-Aldrich (France).

## Results

### mRNA expression of lipid sensing GPCRs in the cerebral cortex of embryonic mice

**Figure 1:**
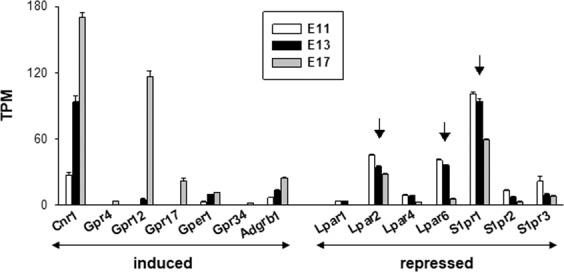
mRNA expression of putative lipid sensing GPCRs in the embryonic murine cortex. The data used to plot this graph were extracted from a previous whole-genome transcriptomic analysis^22^. A total of 60 genes encoding for putative lipid sensing GPCRs were selected (see Table 1). Transcripts of only 14 genes (out of 60) could be detected (e.g. having TPM values ≥ 2). The graph shows the temporal pattern of the mRNA abundance of these 14 genes at 3 embryonic ages: E11, E13 and E17. Genes that were induced (Cnr1, Gpr4, Gpr12, Gpr17, Gper1, Gpr34, Adgrb1) are shown on the left whereas genes that were repressed (Lpar1, Lpar2, Lpar4, Lpar6, S1pr1, S1pr2, S1pr3) appear on the right. Vertical arrows indicate the 3 most abundant transcripts at E13 (except CB_1_, see text for further details).

In order to determine whether NAGly is acting via a GPCR, we analyzed the expression of genes encoding for putative lipid sensing GPCRs in the embryonic cerebral cortex. Table 1 provides the list of the 60 mouse genes selected^26–30^. The transcriptomic data were extracted from a recent RNAseq study^22^. The expression pattern of putative lipid sensing GPCRs was analyzed at 3 embryonic ages: E11, E13 and E17. Only genes for which the number of transcripts per million (TPM) was >2 were considered as significantly expressed^31^, therefore when the number of transcripts was <2 TPM, the gene was eliminated from the analysis. This resulted in the selection of 14 genes encoding for putative lipid sensing GPCRs (Fig. 1). In this RNAseq analysis the genes encoding for GPR18, GPR55 and GPR92, 3 putative targets of NAGly, were not expressed. Overall, the most abundant transcripts were coding for cannabinoid receptors type 1 (CB_1_) (Cnr1 gene), the orphan receptor GPR12, lysophosphatidic acid (LPA) and sphingosine-1 phosphate (S1P) receptors (Fig. 1). Of note, the abundance of CB_1_ and GPR12 transcripts increased markedly during the embryonic development of the cerebral cortex whereas the expression of genes encoding for LPA and S1P receptors was repressed. Since all the live-cell Ca^2+^ imaging reported previously were conducted on cortical cells isolated from E13 brain cerebral cortices^9^ we focused our attention on the most expressed lipid sensing GPCR genes at that embryonic age: S1pr1, Lpar2 and Lpar6 (vertical arrows, Fig. 1). They encode for S1P1, LPA2 and LPA6 receptors, respectively. CB_1_ was excluded from our analysis because NAGly has no affinity for CB_1_ receptors^32^ and the CB_1_ antagonist AM251 did not prevent the NAGly-induced responses in cortical neurons^9^, arguing against a role for these receptors. On the other hand, GPR12 was also not considered as a likely target of NAGly because the GPR12 gene was weakly expressed at E13 (Fig. 1). Its expression was strongly upregulated but only at the end of corticogenesis (E17).

**Table 1:** List of selected 60 murine genes encoding for lipid sensing G protein-coupled receptors (GPCRs).

*EnsemblID*	*Gene name*	*other names*	*Gene description*
ENSMUSG00000044288	Cnr1		cannabinoid receptor 1
ENSMUSG00000062585	Cnr2		cannabinoid receptor 2
ENSMUSG00000046856	Gpr1		G protein-coupled receptor 1
ENSMUSG00000046856	Gpr1		G protein-coupled receptor 1
ENSMUSG00000044317	Gpr4		G protein-coupled receptor 4
ENSMUSG00000046922	Gpr6		G protein-coupled receptor 6
ENSMUSG00000041468	Gpr12		G-protein coupled receptor 12
ENSMUSG00000052229	Gpr17		G protein-coupled receptor
ENSMUSG00000050350	Gpr18		G protein-coupled receptor 18
ENSMUSG00000053647	Gpr30	Gper1	G protein-coupled estrogen receptor 1
ENSMUSG00000071311	Gpr31b		G protein-coupled receptor 31
ENSMUSG00000040229	Gpr34	P2Y12	G protein-coupled receptor 34
ENSMUSG00000026271	Gpr35		G protein-coupled receptor 35
ENSMUSG00000049608	Gpr55		G protein-coupled receptor 55
ENSMUSG00000040372	Gpr63		G protein-coupled receptor 63
ENSMUSG00000021886	Gpr65	TDAG8	G-protein coupled receptor 65
ENSMUSG00000047415	Gpr68	OGR1	G protein-coupled receptor 68
ENSMUSG00000049241	gpr81	Hcar1	hydrocarboxylic acid receptor 1
ENSMUSG00000063234	Gpr84		G protein-coupled receptor 84
ENSMUSG00000051431	Gpr87		G protein-coupled receptor 87
ENSMUSG00000045502	Gpr109A	Hcar2	hydroxycarboxylic acid receptor 2
ENSMUSG00000051209	Gpr119		G-protein coupled receptor 119
ENSMUSG00000064272	Gpr131	Gpbar1	G protein-coupled bile acid receptor 1
ENSMUSG00000021298	Gpr132		G protein-coupled receptor 132
ENSMUSG00000073008	Gpr174		G protein-coupled receptor 174
ENSMUSG00000051212	Gpr183		G protein-coupled receptor 183
ENSMUSG00000034730	Adgrb1	Bai1	adhesion G protein-coupled receptor B1
ENSMUSG00000046908	Ltb4r1		leukotriene B4 receptor 1
ENSMUSG00000040432	Ltb4r2		leukotriene B4 receptor 2
ENSMUSG00000052821	Cysltr1		cysteinyl leukotriene receptor 1
ENSMUSG00000033470	Cysltr2		cysteinyl leukotriene receptor 2
ENSMUSG00000071489	Ptgdr		prostaglandin D receptor
ENSMUSG00000034117	Ptgdr2		prostaglandin D2 receptor 2
ENSMUSG00000019464	Ptger1		prostaglandin E receptor 1
ENSMUSG00000037759	Ptger2		prostaglandin E receptor 2 (subtype EP2)
ENSMUSG00000040016	Ptger3		prostaglandin E receptor 3 (subtype EP3)
ENSMUSG00000039942	Ptger4		prostaglandin E receptor 4 (subtype EP4)
ENSMUSG00000044453	Ffar1		free fatty acid receptor 1
ENSMUSG00000051314	Ffar2		free fatty acid receptor 2
ENSMUSG00000051314	Ffar2		free fatty acid receptor 2
ENSMUSG00000054200	Ffar4		free fatty acid receptor 4
ENSMUSG00000028036	Ptgfr		prostaglandin F receptor
ENSMUSG00000052270	Fpr2		formyl peptide receptor 2
ENSMUSG00000043017	Ptgir		prostaglandin I receptor
ENSMUSG00000038668	Lpar1		lysophosphatidic acid receptor 1
ENSMUSG00000031861	Lpar2		lysophosphatidic acid receptor 2
ENSMUSG00000036832	Lpar3		lysophosphatidic acid receptor 3
ENSMUSG00000049929	Lpar4		lysophosphatidic acid receptor 4
ENSMUSG00000067714	Lpar5		lysophosphatidic acid receptor 5
ENSMUSG00000033446	Lpar6		lysophosphatidic acid receptor 6
ENSMUSG00000044819	Gpr80	Oxgr1, Gpr99, P2Y15	oxoglutarate (alpha-ketoglutarate) receptor 1
ENSMUSG00000056529	Ptafr		platelet-activating factor receptor
ENSMUSG00000050921	P2ry10		purinergic receptor P2Y, G-protein coupled 10
ENSMUSG00000045092	S1pr1		sphingosine-1-phosphate receptor 1
ENSMUSG00000043895	S1pr2		sphingosine-1-phosphate receptor 2
ENSMUSG00000067586	S1pr3		sphingosine-1-phosphate receptor 3
ENSMUSG00000044199	S1pr4		sphingosine-1-phosphate receptor 4
ENSMUSG00000045087	S1pr5		sphingosine-1-phosphate receptor 5
ENSMUSG00000027762	Sucnr1		succinate receptor 1
ENSMUSG00000034881	Tbxa2r		thromboxane A2 receptor

### Presence of functional LPA and S1P receptors

**Figure 2:**
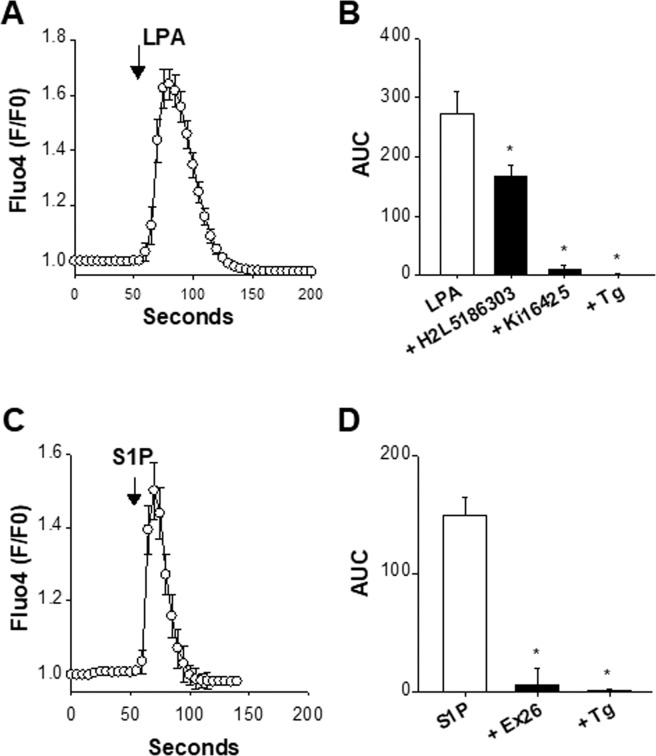
Presence of functional LPA- and S1P-sensitive receptors. The presence of functional LPA and S1P receptors was verified with the fluorescent Ca^2+^ probe Fluo-4. In these experiments, cells were maintained in a nominally Ca^2+^ free saline. Panels A and C show somatic Fluo-4 signals (F/F0) as a function of time in response to 10 µM LPA (n = 10) (panel A) and 10 µM S1P (n = 9) (panel C). Panel B shows the LPA-induced Ca^2+^ rises measured as area under the curve (AUC) in the absence (white bar, n = 10) or presence of H2L5186303 (10 µM, n = 5), Ki16425 (10 µM, n = 7), or after the application of thapsigargin (Tg, 200 nM, n = 5). *p < 0.05 *vs* LPA, one-way ANOVA followed by a Bonferroni’s *post hoc* test. Panel D shows the Fluo-4 responses (measured as area under the curve, AUC) induced by S1P alone (10 µM, n = 9), S1P + Ex26 (1 µM, n = 7), and S1P applied after thapsigargin (Tg, 200 nM, n = 5), with *p < 0.05 *vs* S1P, one-way ANOVA followed by a Bonferroni’s *post hoc* test. Antagonists of LPA and S1P receptors were added 4–7 min before time 0 and remained present throughout the recordings. LPA and S1P can stimulate store-released Ca^2+^. Pre-depleting the ER Ca^2+^ with Tg prevents any response to LPA or S1P.

The activation of S1P and LPA receptors mobilizes Ca^2+^ from internal stores^33–36^. We thus performed live-cell Ca^2+^ imaging fluorescent microscopy experiments with Fluo-4 to assess the presence of functional S1P and LPA receptors. Several cell populations are present in the primary cultures. For instance, 80–85% of the cells express β_III_-tubulin (a marker of post-mitotic neurons) and possess voltage-gated Ca^2+^ channels^37,38^, indicating that most cells display a post-mitotic neuronal phenotype. First, the presence of functional LPA and S1P receptors was assessed in the entire cell population. LPA (10 µM, Fig. 2A) and S1P (10 µM, Fig. 2C) evoked prominent Ca^2+^ rises in ⁓15% (61/416 cells) and ⁓13% (39 out of 303) of the cells tested, respectively. The LPA-induced Ca^2+^ signals were partially blocked by 10 µM H2L5186303, a selective LPA_2/3_ receptor antagonist^39^, and nearly completely suppressed by 10 µM Ki16425, a LPA_1/2/3_ receptor antagonist^39–42^ (Fig. 2B). The percentage of cells responding to LPA was 12% (29/238 cells) and <1% (1/169 cells) with H2L5186303 and Ki16425, respectively. Therefore, H2L5186303 diminished the peak of the Ca^2+^ rise without affecting the number of LPA responsive cells whereas Ki16425 affected both parameters.

The S1P_1_ receptor antagonist Ex26 (1 µM)^43^ reduced the peak amplitude of the S1P-induced Ca^2+^ signals and diminished the number of responsive cells with only 12 cells out 220 tested (⁓5%) generating a Ca^2+^ signal in response to 10 µM S1P (Fig. 2D). In each instance, depleting the ER with thapsigargin prevented the development of a Ca^2+^ rise upon LPA or S1P application (Fig. 2B,D).

Previous reports showed that LPA and S1P receptors are mainly found in proliferative regions of the immature cerebral cortex, with few post-mitotic neurons responding to LPA and S1P^35^. This latter point was checked by using a depolarizing saline solution containing 90 mM KCl to evoke KCl-dependent Ca^2+^ rises. Acutely cultured cells were undifferentiated cells. When cultured for several days, some of these differentiate into neurons (post-mitotic) responding to high-K^+^ whereas non-differentiated cells are not high-K^+^ sensitive. In cultures of embryonic cortical cells, KCl responding cells are identified as neurons whereas KCl-unresponding cells are considered as non-neuronal cells^25^. Overall, only 10 of 67 LPA sensitive cells (⁓15%) generated an intracellular Ca^2+^ rise in response to KCl. These data are consistent with a previous report showing that in the embryonic cerebral cortex LPA receptors are predominantly expressed by neural precursor cells with only a small minority of neurons responding to LPA^35^. On the other hand, 5 of 25 S1P sensitive cells (20%) were KCl-responsive cells. This indicates that the S1P-sensitive cells are also mainly found in KCl-insensitive cells^34^. Taken together, LPA or S1P mobilizes Ca^2+^ from the ER in a subset of cells (<20%). These functional LPA- and S1P-sensitive receptors are essentially expressed by non-neuronal cells^35,44^.

**Figure 3:**
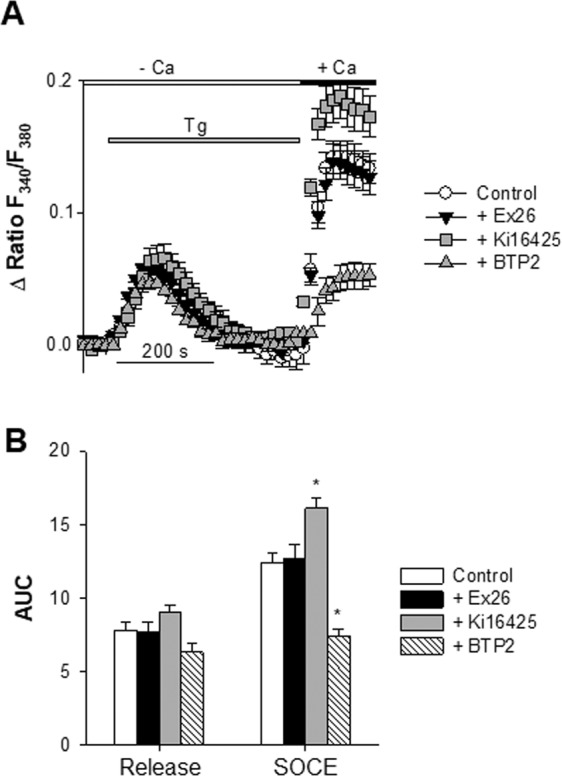
Effects of Ex26, Ki16425, and BTP2 on the thapsigargin-evoked Ca^2+^ release and SOCE. SOCE responses were analysed with Fura-2. Cells were kept in a nominally Ca^2+^-free medium. ER Ca^2+^ stores were depleted with thapsigargin (Tg, 200 nM) before re-introducing external Ca^2+^. The resulting increase in intracellular Ca^2+^ is due to Ca^2+^ entering via the plasma membrane. Panel A shows somatic Ca^2+^ responses (expressed as Δ ratio F340/F380) as a function of time, and generated by the sequential addition of Tg (200 nM, horizontal gray bar) followed by the readmission of 2 mM external Ca^2+^ (horizontal black bar). Four conditions are shown: without antagonists of LPA and S1P receptors (Control, open circles, n = 7), with 1 µM Ex26 (gray triangles, n = 5), with 10 µM Ki16425 (filled squares, n = 5), and with 1 µM BTP2 (symbols, n = 5). When tested, Ex26 (or Ki16425) and BTP2 were added 4–7 and 11–12 min, respectively, before time 0 and were also present during the recordings. One time point out of 3 is shown. Panel B shows the thapsigargin-evoked Ca^2+^ release and SOCE measured as area under the curve (AUC). Mean ± SEM.

Before testing the contribution of LPA and S1P receptors in the NAGly-dependent alteration of SOCE, it was important to check whether the receptor antagonists Ki16425 and Ex26 could alter SOCE on their own. In the following experiments, the ratiometric Ca^2+^ probe Fura-2 was used to analyze SOCE in cells that responded to the KCl challenge (i.e. post-mitotic neurons). Cells, bathed in a nominally Ca^2+^-free medium, were challenged with thapsigargin to deplete ER Ca^2+^ stores. A subsequent re-admission of external Ca^2+^ was followed by an intracellular elevation of Ca^2+^ (open circles, Fig. 3A)^9,24^. This entry of Ca^2+^ was sensitive to the CRAC channel blocker BTP2^45,46^ (1 µM, gray up triangles, Fig. 3A). The thapsigargin-evoked Ca^2+^ release was unaffected by Ex26 (1 µM, filled down triangles) or Ki16425 (10 µM, gray squares) (Fig. 3A). The SOCE response was however upregulated by Ki16425 but not by Ex26. This is further illustrated in Fig. 3B showing the Ca^2+^ release and entry analyzed as area under the curve for each condition tested. Ki16426 enhanced the SOCE signal by nearly 30% (n = 5, p < 0.05) (Fig. 3B, gray bar). Altogether, these data show that the LPA and S1P receptor antagonists used did not alter the ER Ca^2+^ release. The SOCE response was also unaffected by Ex26 but augmented by Ki16426. This potentiating effect was not investigated further.

### NAGly depresses SOCE independently of LPA and S1P receptors

**Figure 4:**
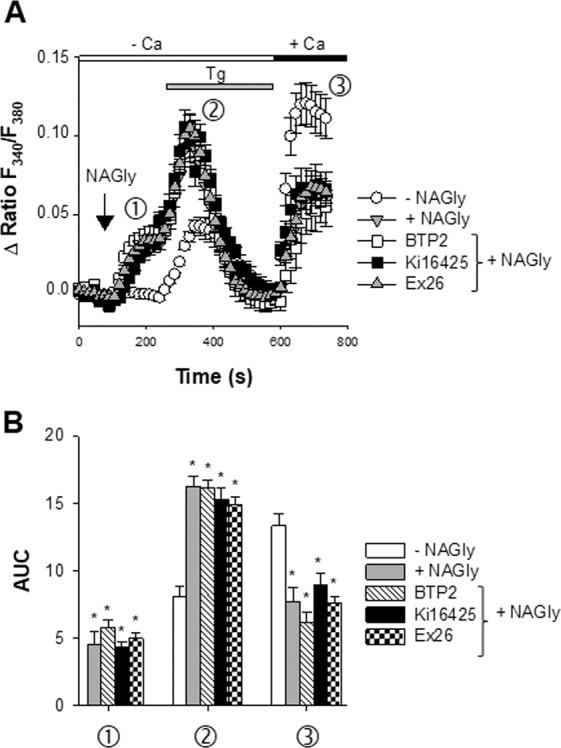
Ex26 and Ki16425 do not reverse the NAGly-induced depression of SOCE. Panel A shows Fura-2 responses (Δ ratio F340/F380) as a function of time before, during the transient application of 200 nM thapsigargin (Tg, horizontal gray bar) to cells kept in a nominally Ca^2+^-free medium (depletion of ER Ca^2+^ stores, phase ➁), and after the readmission of 2 mM external Ca^2+^ (horizontal black bar) (SOCE, phase ➂). Open circles: control conditions (without NAGly) (n = 7). When indicated, 10 µM NAGly was added (vertical arrow) prior to thapsigargin. This elevated the Fura-2 fluorescence (phase ➀) (black triangles, n = 6). Similar experiments were conducted in the presence of NAGly + 10 µM Ki16425 (gray squares, n = 5), NAGly + 10 µM Ex26 (open triangles, n = 4), and NAGly + 1 µM BTP2 (symbol, n = 3). As in Fig. 3, Ki16425 (or Ex26) and BTP2 were added 4–7 and 11–12 min before time 0 and remained present throughout the recordings. One time point out of 3 is shown. Mean ± SEM. Panel B: Area under curve (AUC) measurements of Fura-2 signals under the different conditions tested. Three phases were considered: Ca^2+^ signals prior to the addition of thapsigargin (phase ➀), the thapsigargin-induced Ca^2+^ release (phase ➁) and SOCE (phase ➂). *p < 0.05 *vs* NAGly-untreated cells, one-way ANOVA followed by a Bonferroni’s *post hoc* test.

After having shown the presence of functional receptors sensitive to LPA and S1P, their involvement in the NAGly-induced impairment of SOCE was considered. In the following set of experiments, Fura-2-loaded cells were first stimulated with a K^+^-rich saline (90 mM KCl) before recording SOCE responses in neurons (i.e. in KCl-responsive cells). Figure 4A shows SOCE without NAGly (open circles) and in the presence of NAGly (10 µM, gray down triangles). As already illustrated^9^, NAGly exerts complex actions on neuronal Ca^2+^ signalling: (i) it induces a release of cations (Ca^2+^ and Zn^2+^) that develops prior to thapsigargin addition (phase ➀, Fig. 4A); (ii) it upregulates the thapsigargin-dependent Ca^2+^ release (phase ➁); and (iii) reduces the amplitude of SOCE (phase ③). Even in the presence of 1 µM Ex26 (gray up triangles) or 10 µM Ki16425 (filled squares, Fig. 4A), NAGly elevated the Fura-2 fluorescence on its own (phase ➀) and potentiated the thapsigargin-evoked Ca^2+^ release (phase ➁). The NAGly-induced inhibition of SOCE (phase ③) was also not affected by Ex26 or Ki16425 (Fig. 4A). NAGly had however no inhibitory action on the entry of Ca^2+^ when added together with BTP2 (open squares, Fig. 4A).

The Fura-2 Ca^2+^ signals (phases ➀, ➁, ③) observed without NAGly (white bars), with NAGly (gray bars), NAGly + Ki16425 (black bars), NAGly + Ex26 (black/white bars), and NAGly + BTP2 (hatched bars) were analyzed as area under curve (AUC) (Fig. 4B). In conclusion, Ki16425 and Ex26 failed to affect the NAGly-evoked Ca^2+^ rise (phase ➀). These blockers also did not influence the potentiation of the thapsigargin-induced Ca^2+^ signal induced by NAGly (phase ➁) and the NAGly-dependent depression of SOCE (phase ③). It is proposed that NAGly inhibits a BTP2-sensitive Ca^2+^ entry pathway without recruiting LPA or S1P-sensitive receptors.

## Discussion

NAGly inhibits SOCE^20^. This impairment has been observed in every cell type and cell line tested so far like fibroblasts, neurons, EA.hy926 (human endothelial cell line), INS-1 832/13 (rat pancreatic β-cell line), and RBL-2H3 cells (rat basophilic leukemia cell line)^9,20,21^. However, the mechanism by which NAGly alters SOCE is unclear. In the present study we addressed the question of the contribution of lipid sensing GPCRs as targets of NAGly with the aim to gain a better knowledge on neuronal SOCE functioning and regulation. To reach that goal, we took advantage of a recent transcriptomic analysis of the whole murine genome by RNA-seq.^22^. This allowed us to consider the mRNA expression of 60 putative lipid sensing GPCRs^26–30^. Overall, transcripts of 14 genes (⁓25%) were detected. Their abundance varied during embryonic development with 7 genes being induced (Cnr1, Gpr4, Gpr12, Gpr17, Gper1, Gpr34, Adgrb1) and 7 genes being repressed (Lpar1, Lpar2, Lpar4, Lpar6, S1pr1, S1pr2, S1pr3). At E13, age at which cerebral cortices were collected to perform the Ca^2+^ imaging experiments^9^, the most abundant mRNAs were those coding for CB_1_ and S1P_1_ receptors, followed by LPA_2_ and LPA_6_ receptors. Since the cannabinoid receptor CB_1_ does not seem to mediate the NAGly-dependent impairment of SOCE^9^, only the contribution of S1P and LPA receptors in the NAGly-mediated modulation of SOCE was investigated.

Five subtypes of S1P receptors are known (S1P_1–5_). They belong to the group of GPCRs and mediate most of the biological actions of the bioactive sphingolipid S1P^30^. Embryonic cerebral cortices displayed a high mRNA level of S1P_1_ receptors that declined during embryonic brain development. In addition, cultured cortical cells expressed functional receptors coupled to the release of Ca^2+^ from the ER and sensitive to the S1P_1_ antagonist Ex26. These findings are in line with previous reports showing that S1P_1_ is the major S1P receptor of the murine embryonic brain, followed by S1P_2_ and S1P_3_ receptors. It is detected as early as E14, highly expressed in proliferative regions (neurogenic ventricular zone) but its expression decreases at E16 and E18^47^. The activation of S1P_1_ receptors is coupled to the mobilization of Ca^2+^ ^33^.

LPA receptors constitute another important family of GPCRs sensitive to bioactive lipids^30,39^. LPA signalling is of particular physiological relevance for the embryonic brain cortex^48^. At E12.5 the most abundant transcripts in the telencephalon are LPA_1_, LPA_2_ and LPA_4_^35^. In the present work, the main genes expressed at E13 were encoding for LPA_2_ and LPA_6_. Moreover, the application of LPA caused the release of Ca^2+^ from the ER. These responses were highly sensitive to the LPA_1/3_ antagonist Ki16425 but moderately affected by the LPA_2/3_ antagonist H2L5186303^30,39^. The pharmacological dissection of the LPA-induced Ca^2+^ signalling pointing to LPA_1/3_ as the likely receptors responding to LPA is difficult to reconcile with the gene analysis showing that LPA_1_ and LPA_3_ are, respectively, very weakly expressed and undetected. The pharmacological properties of native LPA receptors of cortical neurons may differ from those of LPA receptors heterogeneously expressed.

After having shown the presence of functional LPA and S1P receptors, their contribution to the NAGly-dependent depression of SOCE was evaluated. The pharmacological blockade of S1P and LPA receptors with Ex26 or Ki16425 did not abolish or attenuate the NAGly-dependent impairment of SOCE. Some cellular responses of NAGly have been shown to be mediated by the orphan receptor GPR55^11^. However, we found no evidence for the presence of significant levels of GPR55 mRNA. Furthermore, the GPR55 agonist AM251^49^, which induces a GPR55-dependent mobilization of Ca^2+^ with an EC_50_ of ~0.6 µM^50^, fails to evoke any Ca^2+^ release when applied to cortical cells at 10 µM. This further suggests that GPR55 does not participate in the NAGly-induced alteration of neuronal Ca^2+^ signalling.

In conclusion, our data show that NAGly inhibits a BTP2-sensitive Ca^2+^ entry, which is most likely a SOCE. This occurs independently of GPR55, LPA and S1P receptors (present report), and via a mechanism insensitive to the pertussis toxin^9^. It is worth recalling that NAGly regulates voltage-gated Ca^2+^ channel activity without acting on GPCRs^8,13^. Although we cannot exclude the possibility that NAGly acts on an orphan lipid sensing GPCR that was not considered in the present study, our report suggests that NAGly disturbs the coupling of the core components of the SOCE machinery (STIM-Orai)^20^. This inhibitory process does not seem to develop in response to an intracellular signalling cascade. These past^9^ and present data show that the phytocannabinoid cannabidiol, the endocannabinoid AEA and its derivative NAGly are potent inhibitors of neuronal SOCE. This indicates that NAGly and endocannabinoids are endogenous SOCE modulators, and raises the possibility that the depression of SOCE could play a role in the neuro-behavioural effects of cannabinoids and signalling lipids.
